# The impact of noradrenergic neurotoxin DSP-4 and noradrenaline transporter knockout (NET-KO) on the activity of liver cytochrome P450 3A (CYP3A) in male and female mice

**DOI:** 10.1007/s43440-022-00406-8

**Published:** 2022-08-26

**Authors:** Ewa Bromek, Przemysław Jan Danek, Jacek Wójcikowski, Agnieszka Basińska-Ziobroń, Renata Pukło, Joanna Solich, Marta Dziedzicka-Wasylewska, Władysława Anna Daniel

**Affiliations:** 1 Department of Pharmacokinetics and Drug Metabolism, Maj Institute of Pharmacology, Polish Academy of Sciences, Smętna 12, 31-343 Kraków, Poland; 2 Department of Pharmacology, Maj Institute of Pharmacology, Polish Academy of Sciences, Smętna 12, 31-343 Kraków, Poland

**Keywords:** Cytochrome P450, Liver, Brain, Noradrenergic system, Neuroendocrine regulation, DSP-4 neurotoxin, NET knockout

## Abstract

**Background:**

Our earlier studies have shown that the brain noradrenergic system regulates cytochrome P450 (CYP) in rat liver via neuroendocrine mechanism. In the present work, a comparative study on the effect of intraperitoneal administration of the noradrenergic neurotoxin DSP-4 and the knockout of noradrenaline transporter (NET-KO) on the CYP3A in the liver of male and female mice was performed.

**Methods:**

The experiments were conducted on C57BL/6J WT and NET^–/–^ male/female mice. DSP-4 was injected intraperitoneally as a single dose (50 mg/kg ip.) to WT mice. The activity of CYP3A was measured as the rate of 6β-hydroxylation of testosterone in liver microsomes. The CYP3A protein level was estimated by Western blotting.

**Results:**

DSP-4 evoked a selective decrease in the noradrenaline level in the brain of male and female mice. At the same time, DSP-4 reduced the CYP3A activity in males, but not in females. The level of CYP3A protein was not changed. The NET knockout did not affect the CYP3A activity/protein in both sexes.

**Conclusions:**

The results with DSP-4 treated mice showed sex-dependent differences in the regulation of liver CYP3A by the brain noradrenergic system (with only males being responsive), and revealed that the NET knockout did not affect CYP3A in both sexes. Further studies into the hypothalamic–pituitary–gonadal hormones in DSP-4 treated mice may explain sex-specific differences in CYP3A regulation, whereas investigation of monoaminergic receptor sensitivity in the hypothalamic/pituitary areas of NET^–/–^ mice will allow for understanding a lack of changes in the CYP3A activity in the NET-KO animals.

**Supplementary Information:**

The online version contains supplementary material available at 10.1007/s43440-022-00406-8.

## Introduction

Cytochrome P450 (CYP) enzymes are members of the superfamily of heme-containing monooxygenases. CYP isoforms are involved in the metabolism of high-activity endogenous substrates (e.g., steroids, arachidonic acid, neurotransmitters, and vitamins), most of clinically important drugs and harmful xenobiotics [1]. The CYP3A subfamily is the most abundant in human liver and belongs to the most expressed subfamilies in animals. There are many known drugs which are substrates for CYP3A, including: psychotropics, calcium channel blockers (e.g., nifedipine), cyclosporine, macrolide antibiotics (e.g., erythromycin), antifungal drugs (e.g., clotrimazole, ketoconazole), and endogenous substances, mainly steroids (testosterone, progesterone, estradiol, and androstenedione), as well as bile acids and retinol. Regulation of CYP3A gene expression in humans is one of the best known among the CYP genes. It has been shown that the regulation of CYP3A expression involves different nuclear receptors, such as GR, PXR, CAR and VDR [2–5]. In addition, some nuclear factors, such as HNF-4α (CYP3A4), HNF-4 and USF-1 (CYP3A23) are likely needed for optimal CYP3A expression. The CYP3A subfamily in rodents consist of different enzymes in rats (CYP: 3A1/3A23, 3A2, 3A9, 3A18, 3A62) and in mice (CYP: 3A11, 3A13, 3A16, 3A25, 3A41, 3A44) [6].

Many hormones, such as the growth hormone, thyroid hormones (T_3_ and T_4_), glucocorticoids and sex hormones play a substantial role in the physiological regulation of the cytochrome P450 expression in the liver via activation of nuclear/cytoplasmic receptors. In this way, they regulate the transcription of genes encoding CYP enzymes [7–12]. Our previous study has clearly indicated that the dopaminergic [13], noradrenergic [14–16] and serotonergic [17] systems of the brain are involved in the neuroendocrine regulation of liver CYP.

DSP-4 is a neurotoxin specific to noradrenergic nerve terminals that crosses the blood–brain barrier and induces a selective long-term depletion of noradrenaline in the brain [18]. Noradrenaline/norepinephrine transporter (NET) in neuronal plasma membrane is responsible for recapturing the released noradrenaline back into the presynaptic cell to maintain homeostasis of the neurotransmitter system. It is also one of the main targets of psychoactive drugs. The NET knockout mice have been used to study the neurochemical background of mental disorders and the mechanism of action of antidepressants and psychostimulants [19–23]. The NET knockout leads to a decrease in the tissue level of NE in the brain, so it constitutes an interesting experimental model allowing for comparison of the effect of acute NE depletion produced by a single DSP-4 dose with that evoked by NET gene knockout.

Our earlier studies conducted on male rats have shown that the brain monoaminergic neurotransmitters are able to change CYP enzyme activity and expression. The selective noradrenaline toxin DSP-4 given intraperitoneally or intracerebrally (injected to the brain lateral ventricles or to the hypothalamic paraventricular or arcuate nuclei) evoked significant changes in the activity of CYP in rat liver [14, 15, 18]. Furthermore, another neurotoxin 6-OHDA given into the locus coeruleus also induced alterations in the protein level and activity of CYP enzymes in the rat liver [16]. The effects produced by the applied neurotoxins in male rats depended on the route of administration (intraperitoneal vs. intracerebral) and the site of neurotoxin injection in the brain, indicating a complex neuroendocrine mechanism of hepatic cytochrome P450 regulation by the brain noradrenergic system.

In the present work, we aimed to perform a comparative study on the effect of intraperitoneal DSP-4 administration on the CYP3A activity and protein level in the liver of male and female mice to observe possible species- or sex-dependent differences in the regulation of this enzyme in rodents. Moreover, we were interested in finding out whether the knockout of noradrenaline/norepinephrine transporter (NET-KO), an important regulator of monoamine homeostasis can affect that enzyme.

## Materials and methods

### Animals

Heterozygous NET^+/–^ mice of C57BL/6J background came from Dr. M. Caron (Duke University, Medical Center, Durham, NC, USA). They were mated to get homozygous NET^+/+^ and NET^–/–^ mice as reported by Xu et al. [19]. Both sexes were used. The genotypes were confirmed by PCR and application of the primers mNETEx2s (5ʹ-GCT TTA TGG CATGTA GTG TGC AC-3ʹ), mNETEx2as (5ʹ-GCT TTCTGC TTG AAC TTG AAG GC-3ʹ), and EGFP as (5ʹ-GCC GGA CAC GCTGAA CTT GTG-3ʹ) to amplify a 700 and 500 bp PCR product in the case of NET^+/+^ and NET^–/–^ mice, respectively. The NET^+/+^ mice served as a control group, and were compared with the knockout (NET^–/–^) mice of the same sex. The control and knockout groups (male and female groups) comprised 12 mice. The mice had free access to laboratory food and tap water and were held at a constant ambient temperature (24 °C) under 12 h light/dark cycle. Animals were maintained pursuant to the guidelines of the European Union Directive 86/609/EWG and were kept in line with the decision of the Minister of Environment (no. 01-49/2009). In the DSP-4 experiment, we used NET^+/+^ male and female mice of C57BL/6J background. There were 8 mice in the control (saline) and 8 animals in DSP-4 groups.

### Drugs and treatment

*N*-(2-Chloroethyl)-*N*-ethyl-2-bromobenzylamine (DSP-4), NADP, glucose-6-phosphate-dehydrogenase, glucose-6-phosphate, and noradrenaline (NA) were delivered by Sigma (St. Louis, USA). Testosterone and its metabolites were purchased from Steraloids (Newport, KY, USA). Solutions were prepared on the day of experiment. DSP-4 was dissolved in isotonic saline and was injected intraperitoneally as one dose (50 mg/kg ip.) [18]. Control mice received saline. For Western blotting, polyclonal anti-CYP3A4 antibody from Fine Test (Wuhan, Hubei, China), polyclonal anti-β-actin antibody from Sigma (St. Louis, MO, USA), and rat cDNA expressed CYP3A1 and CYP3A2 enzymes (Supersomes) from Gentest Corp. (Woburn, MA, USA) were used.

### Measurement of brain neurotransmitters and their metabolites by HPLC

The brain tissue levels of noradrenaline (NA), dopamine (DA) and its two metabolites: 3,4-dihydroxyphenylacetic acid (DOPAC), and homovanillic acid (HVA), and serotonin (5-HT), with its metabolite 5-hydroxyindoleacetic acid (5-HIAA) were assessed using ultra-high-performance liquid chromatography (UHPLC) with coulochemical detection using earlier described method with minor modifications [24, 25]. Brain tissue was homogenized by sonification in 20 volumes (v/w) of ice-cold 0.1 M perchloric acid (HClO_4_). Homogenates were centrifuged at 15,000×*g* for 15 min at 4 °C. The obtained supernatants transferred to new Eppendorf tubes were centrifuged at 15,000×*g* for 5 min at 4 °C and were filtered through a 0.2 μm membrane filter. The final samples were kept at − 80 °C until further analyzed. Subsequently, 10 μl aliquots were injected into the UHPLC Ultimate 3000 system Dionex (Thermo Scientific, Germering, Germany). The used system consisted of ECD-3000RS electrochemical detector, 6011RS ultra coulometric analytical cell, WPS-3000RS autosampler and a Hypersil Gold analytical column 3 µm, 100 × 3 mm (Thermo Scientific, Waltham, MA, USA). Neurotransmitters and their metabolites were eluted using the mobile phase containing: KH_2_PO_4_ (0.1 M), EDTA (0.5 mM), sodium 1-octanesulfonate (80 mg/l), methanol (4%), adjusted to pH = 4.0 with 85% H_3_PO_4_, at the flow rate of 0.6 ml/min and the column temperature of 30 °C. The potentials of coulometric cell were: E1 = − 50 mV, E2 = +350 mV [25]. The identification and quantification of the chromatographic peaks were performed by comparing with the reference peaks of standards: NA, DA, 5-HT, DOPAC and 5-HIAA (Sigma) at a concentration of 50 ng/ml and HVA (Sigma) at a concentration of 100 ng/ml [24]. The data were analyzed and processed using Chromeleon 7 software (Thermo Scientific, Waltham, MA, USA) [25]. The detection limit for noradrenaline, dopamine, DOPAC, HVA and 5-HIAA was 0.5 pg/10 μl, and for serotonin was 1 pg/10 μl.

### Preparation of liver microsomal fraction

Liver microsomes were prepared from WT (NET^+/+^), DSP-4-treated WT (7 days after DSP-4 administration), and NET^–/–^ male and female mice. Microsomes were isolated from individual mouse livers by homogenization and differential centrifugation (11,000×*g* and 100,000×*g*) in a 20 mM Tris/KCl buffer (pH 7.4). The obtained microsomes were washed with 0.15 M KCl [26].

### Determination of CYP3A activity and enzyme protein level

The activity of mouse CYP3A was studied by measuring the rate of CYP3A-specific reaction, i.e., the testosterone 6β-hydroxylation, at a testosterone concentration of 100 μM using the HPLC with UV detection [27].

Western blot was carried out as described previously [14]. Briefly, microsomal proteins (10 µg) were applied on SDS gels in a Laemmli buffer system. After separation, they were transferred onto a nitrocellulose membrane, probed with primary antibodies and incubated with the appropriate horseradish peroxidase-conjugated secondary antibodies. The CYP3A bands were visualized by enhanced chemiluminescence. Rat cDNA-expressed CYP3A1 and CYP3A2 (1 µg) were used as standards (Supersomes). β-Actin immunoreactivity was used for normalization of the obtained data.

### Measurement of pituitary growth hormone releasing hormone (GHRH)

The pituitary level of GHRH was measured in control and DSP-4 treated male and female mice using the mouse GHRH ELISA kit from Fine Test (Wuhan, Hubei, China). Absorbance was measured using a Synergy Mx Monochromator-Based Multi-Mode Microplate Reader (Biotek, Winooski, VT, USA).

### Statistical analysis

**Fig. 1:**
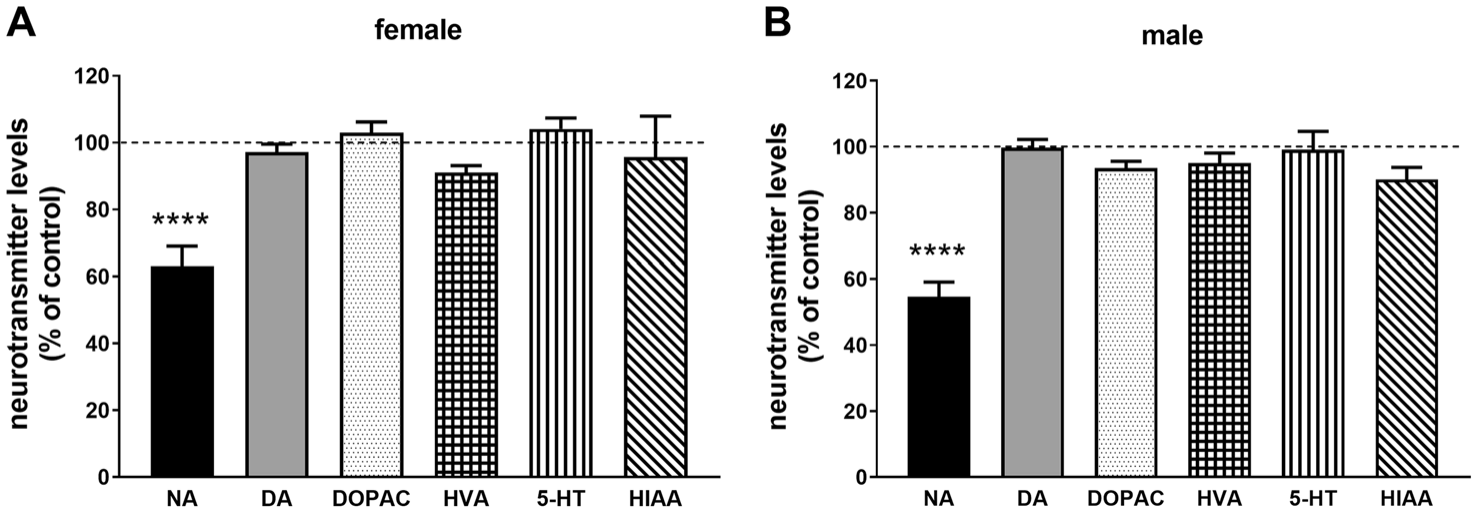
The influence of DSP-4 administration (50 mg/kg ip.) on the brain level of neurotransmitters in female (**A**) and male (**B**) mice. All values are shown as the mean ± SEM (*n* = 7–8). The results were evaluated statistically using Student’s *t* test: ****p < 0.0001, *t* = 9.047, df = 14 (females) and ****p < 0.0001, *t* = 5.839, df = 14 (males), compared to the control of the same sex. The mean numerical values and raw numerical data of neurotransmitter levels for each group are presented in the Table 1 of Supplementary materials

**Fig. 2:**
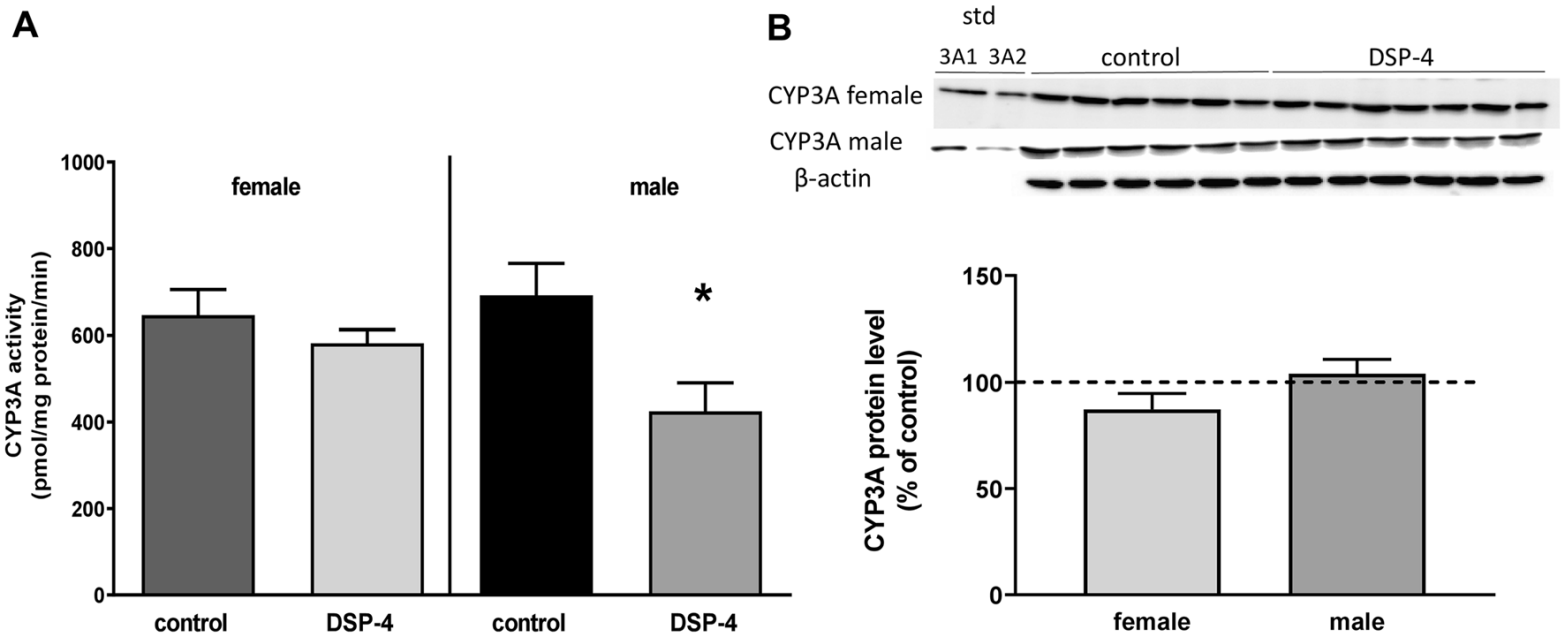
The effect of DSP-4 administration (50 mg/kg ip.) on the CYP3A activity (**A**) and protein level (**B**) in the liver of male and female mice. All values are shown as the mean ± SEM (*n* = 7–8). The results were evaluated statistically using Student’s *t *test: *p < 0.05, *t* = 2.672, df = 13, compared to the control of the same sex. *std* standard. The mean numerical values and raw numerical data of enzyme activity for each group are presented in the Table 2 of Supplementary materials

The male and female groups were investigated separately. All data had a normal distribution (Shapiro–Wilk test and Kolmogorov–Smirnov test). The results were analyzed statistically using Student’s *t* test. All the obtained values are the mean ± SEM from 7 to 8 (DSP-4 mice) or 9–12 (NET-KO mice) animals. The statistical reports (*p*, *t* and df-values) for statistically significant results have been included in figure captions (Figs. 1 and 2).

## Results and discussion

This is the first report on the CYP3A activity investigated in DSP-4-treated and NET knockout male and female mice. The results indicate sex-dependent differences in the effect of the neurotoxin. They show that intraperitoneal administration of DSP-4 decreases the CYP3A activity in males, but not in females, while NET knockout does not affect the CYP3A activity, neither in males nor in females.

DSP-4 injected intraperitoneally to WT mice evoked a selective and significant decrease in the noradrenaline level in the brain of both male and female mice (down to 54.6% and 63.1%, respectively). The levels of dopamine and serotonin and their metabolites remained unchanged (Fig. 1A, B). At the same time, DSP-4 produced reduction in the CYP3A activity measured as the rate of testosterone 6β-hydroxylation in male, but not in female mice (Fig. 2A), while the level of CYP3A protein did not change (Fig. 2B).

Since the elimination of DSP-4 from the periphery is faster than from the brain and peripheral noradrenergic nerves are more resistant to the neurotoxin [28], it can be assumed that the neurotoxin was practically removed from the periphery 7 days after its single administration and it had no significant effect on peripheral NE level at the time of the experiment. Jaim-Etcheverry and Zieher [29] showed that the systemic injection of DSP-4 to adult rats transiently altered sympathetic neurons in the periphery, but in the central nervous system, the compound produced a marked and prolonged reduction of noradrenaline levels in all brain regions. When a single dose of DSP-4 (50 mg/kg) was given intraperitoneally to rats, a quicker restoration of noradrenaline concentration was observed in peripheral organs than in the brain. The noradrenaline level in the cerebral cortex was markedly reduced (down to 20% of the control), whereas only a slight decline in noradrenaline level was found in peripheral organs (to 80% of the control) 7 days after injection. The above data suggest that the effect of DSP-4 on liver CYP3A observed in our experiment 7 days after a single intraperitoneal dose should rather be ascribed to a decrease in brain noradrenaline level.

Such results, i.e., a significant decrease in the CYP3A activity, but not protein, were also observed in our previous study after intraperitoneal administration of DSP-4 to male rats [18]. It seems possible that a slower degradation of CYP3A protein (as a result of unspecific effect of the neurotoxin) along with a simultaneous decrease in CYP3A expression/synthesis (as previously observed in rats) may yield no change in the enzyme protein level as a resultant of the two mentioned processes. However, if DSP-4 was injected to the lateral brain ventricles, a concurrent decrease in the CYP3A activity, protein level and mRNA was noticed [14]. Similar effect was found when DSP-4 was injected to the hypothalamic arcuate nuclei producing growth hormone releasing hormone (GHRH), while an opposite effect (i.e., a parallel increase in the CYP3A activity, protein level and mRNA) was observed when the neurotoxin was injected to the hypothalamic paraventricular nuclei producing somatostatin or to the locus coeruleus innervating the paraventricular nuclei [15, 16]. In all those cases, the CYP3A expression and activity were positively correlated with serum concentration of growth hormone (GH). It is noteworthy that after intraventricular injection of DSP-4 to male rats, a parallel decrease in serum concentration of testosterone, GH and the CYP3A expression/activity was observed [14]. The results of the above studies suggest that the brain noradrenergic system regulates CYP3A via GH and testosterone.

**Fig. 3:**
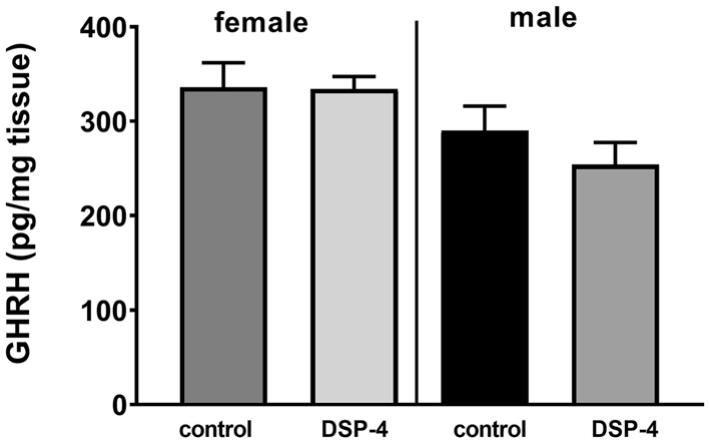
The pituitary level of growth hormone releasing hormone (GHRH) in control and DSP-4-treated male and female mice. All values are shown as the mean ± SEM (*n* = 7–8); the results were evaluated statistically using Student’s *t* test (no significance was found). The mean numerical values and raw numerical data of enzyme activity for each group are presented in the Table 3 of Supplementary materials

GH secretion is controlled by the hypothalamic peptides—positively by GHRH and ghrelin and negatively by somatostatin. Therefore, it seems that the observed difference between male and female mice in the effect of DSP-4 may be connected with different patterns of the GH secretion which is pulsatile in males and more continuous in females [7, 8, 30]. Studies in mice indicate that somatostatin is important for masculinized GH secretion, but does not affect female GH secretion pattern [31]. The results of our study showed a tendency towards a decrease in the pituitary GHRH level in male, but not in female mice treated with DSP-4 (Fig. 3). Those sex-specific GH secretion patterns are regulated hormonally by testosterone and estrogens. Sex steroids androgens and estrogens can modulate GH action both centrally via their effect on GH secretion and peripherally by modifying GH responsiveness [32, 33]. Some evidence suggests that testosterone-induced stimulation of GH secretion is partly dependent on the aromatization of testosterone to estradiol [34, 35]. However, in rodents estrogen represses hypothalamic GHRH and pituitary GHRH receptor gene expression [32]. The regulation of GH secretion and cytochrome P450 expression/activity by sex hormones seems very complex and needs further investigation. It is conceivable that brain noradrenaline differently modulates gonadotropin releasing hormones in males and females and the regulation of GH secretion via testosterone and somatostatin predominates in males. Further studies into the hypothalamic, pituitary and gonadal hormones in DSP-4 treated mice may explain sex-related differences in CYP3A regulation by the brain noradrenergic system.

On the other hand, in mice with selective ablation of glucocorticoid receptors in the noradrenergic system (GR^DBHCre^ mice), the effect on the activity of CYP3A was observed only in females [36], though brain noradrenaline was slightly diminished in male mice only [37]. Anyway, the results of this experiment also indicate sex-specific differences in CYP3A regulation by brain noradrenaline in rodents, and suggest that in females this regulation may be more dependent on corticosterone than on growth hormone, while for males it is the opposite [2, 38].

**Fig. 4:**
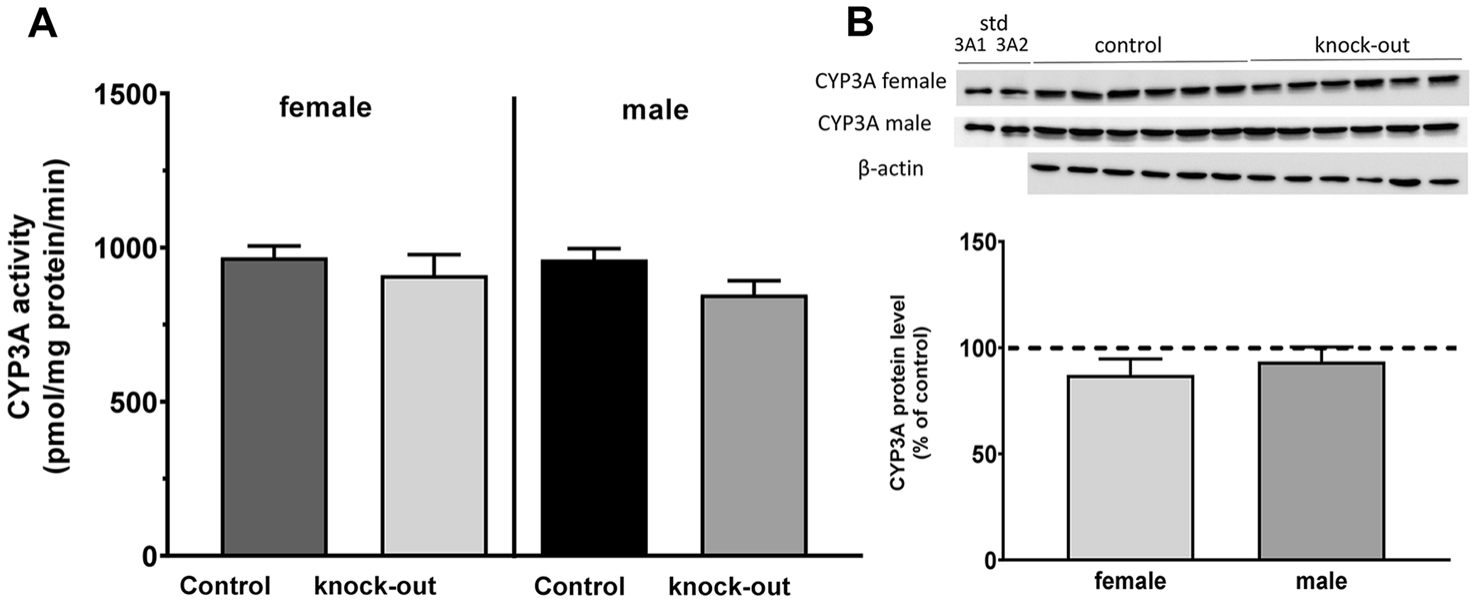
The CYP3A activity (**A**) and protein level (**B**) in NET^+/+^ (control) and NET^–/–^ (NE knockout) male and female mice. All values are shown as the mean ± SEM (*n* = 9–12); the results were evaluated statistically using Student’s *t* test (no significance was found). *std* standard. The mean numerical values and raw numerical data of enzyme activity for each group are presented in the Table 4 of Supplementary materials

In another experiment with interference in the noradrenergic system, i.e., using noradrenaline transporter knockout mice, no change in the CYP3A activity and protein level was observed, neither in males nor in females (Fig. 4). NET^–/–^ mice show reduced intraneuronal neurotransmitter level, but elevated extracellular noradrenaline concentration [19, 22, 39], which leads to adaptive responses in brain neurotransmitter systems, such as downregulation of α_1_-receptors and presynaptic dopamine receptors, and supersensitivity of postsynaptic D_2_/D_3_ receptors [19, 20]. As a consequence, the NET^–/–^ mice are supersensitive to psychostimulants and show depression-resistant behavioral and biochemical responses [19–21, 40, 41]. Therefore, the NET knockout mice constitute an interesting experimental model allowing for comparison of the effect of acute NE depletion produced by a single DSP-4 dose with that evoked by NET gene knockout. As our study shows, there is a difference between results obtained in these two models. The NET knockout did not affect the CYP3A activity in the liver in both sexes, probably due to the abovementioned adaptation processes in neurotransmitter systems.

It is suggested that noradrenaline stimulates somatostatin secretion via α_1_-receptor activation in the paraventricular nuclei and enhances GHRH secretion from the arcuate nuclei via α_2_-receptor activation which results in either inhibition or stimulation of GH release in the pituitary, respectively [42–44]. Activation of β-receptor reduces GH secretion via stimulation of somatostatin release in humans and sheep [45–47]. A detailed study into the functioning of monoaminergic receptors in the hypothalamic paraventricular and arcuate nuclei of NET^–/–^ male and female mice could explain the observed sex-dependent regulation of liver cytochrome P450 by the brain noradrenergic system.

Understanding the mechanism of sex-dependent regulation of liver cytochrome P450 by noradrenergic system is important in personalized therapy with drugs acting via adrenergic mechanisms, since apart from their therapeutic action, they may also impact on the cytochrome P450 expression/activity and metabolism of drugs or endogenous substrates. Adrenergic receptors are involved in physiological/pharmacological regulation of the function of different body systems, including the nervous, endocrine, cardiovascular and respiratory systems. Hypertension is a common disease treated with α_2_ noradrenergic receptor agonists, α_1_-receptor antagonists or β_1_-blockers, while β_2_-agonists are used as bronchodilators. The knowledge of neuroendocrine regulation of cytochrome P450 may help to predict changes in the activity of cytochrome P450 and CYP-mediated metabolism in the liver of both sexes during pharmacotherapy.

## Conclusions

The results in DSP-4 treated mice showed sex-dependent differences in the regulation of liver CYP3A by the brain noradrenergic system and revealed that the NET knockout did not affect CYP3A in both sexes. Further studies into the hypothalamic–pituitary–gonadal hormones in DSP-4-treated mice may explain sex-related differences in CYP3A regulation, whereas investigation of monoaminergic receptor sensitivity in the hypothalamic/pituitary areas of NET^–/–^ mice will allow for understanding a lack of changes in the CYP3A activity in the NET-KO animals.

## Supplementary Information

Below is the link to the electronic supplementary material.

Supplementary file1 (DOCX 19 KB)

Supplementary file2 (TIF 351 KB)

## Data Availability

All data generated or analyzed during this study are included in this published article and its supplementary information files.
