# Supplementary material for: The impact of noradrenergic neurotoxin DSP-4 and noradrenaline transporter knockout (NET-KO) on the activity of liver cytochrome P450 3A (CYP3A) in male and female mice
Source: Pharmacol Rep. 2022 Aug 26;74(5):1107–14. doi: 10.1007/s43440-022-00406-8 (PMC9584982; doi:10.1007/s43440-022-00406-8)
Supplement: Supplementary file 1 — Supplementary file1 (DOCX 19 KB) [file 43440_2022_406_MOESM1_ESM.docx]

**Supplementary Data**

**Table 1. Brain neurotransmitter levels in control and DSP-4 treated female and male mice.**

| Neurotransmitters  (pg/mg tissue) | **females** | | **males** | |
| --- | --- | --- | --- | --- |
|  | control | DSP-4 | control | DSP-4 |
| **Noradrenaline** | 318 | 172 | 367 | 167 |
|  | 305 | 125 | 359 | 192 |
|  | 343 | 258 | 342 | 158 |
|  | 290 | 173 | 387 | 161 |
|  | 294 | 191 | 335 | 164 |
|  | 302 | 278 | 324 | 163 |
|  | 289 | 163 | 339 | 233 |
|  | 290 | 171 | 318 | 277 |
| Mean + SEM | **304 + 7** | **191 + 18** | **346 + 8** | **189 + 15** |
| **Serotonin** | 270 | 290 | 251 | 284 |
|  | 222 | 267 | 210 | 265 |
|  | 267 | 362 | 249 | 265 |
|  | 244 | 241 | 313 | 253 |
|  | 233 | 235 | 265 | 205 |
|  | 258 | 240 | 230 | 109 |
|  | 215 | 241 | 235 | 204 |
|  | 213 | 236 | 232 | - |
| Mean + SEM | **240 + 8** | **264 + 16** | **248 + 11** | **226 + 23** |
| **Dopamine** | 723 | 834 | 737 | 740 |
|  | 734 | 736 | 688 | 710 |
|  | 876 | 753 | 732 | 790 |
|  | 701 | 671 | 871 | 774 |
|  | 712 | 750 | 792 | 677 |
|  | 746 | 686 | 679 | 660 |
|  | 743 | 704 | 695 | 785 |
|  | 825 | 755 | 734 | 773 |
| Mean + SEM | **757 + 21** | **736 + 18** | **741 + 23** | **739 + 18** |
| **HVA** | 83 | 87 | 82 | 87 |
|  | 93 | 83 | 88 | 72 |
|  | 117 | 87 | 86 | 83 |
|  | 87 | 82 | 88 | 93 |
|  | 90 | 91 | 92 | 80 |
|  | 96 | 87 | 91 | 80 |
|  | 98 | 88 | 97 | 95 |
|  | 110 | 100 | 80 | 79 |
| Mean + SEM | **97 + 4** | **88 + 2** | **88 + 2** | **84 + 3** |
| **DOPAC** | 85 | 83 | 81 | - |
|  | 67 | 80 | 83 | 77 |
|  | 87 | 97 | 83 | 80 |
|  | 82 | 78 | 100 | 81 |
|  | 87 | 88 | 84 | 78 |
|  | 89 | 96 | 81 | - |
|  | 94 | 90 | 94 | 87 |
|  | 97 | 97 | 92 | 86 |
| Mean + SEM | **86 + 4** | **89 + 3** | **88 + 3** | **82 + 2** |
| **HIAA** | 104 | 102 | 97 | 93 |
|  | 93 | 104 | 97 | 79 |
|  | 108 | 162 | 91 | 78 |
|  | 101 | 95 | 115 | 94 |
|  | 108 | 77 | 101 | 95 |
|  | 85 | 42 | 110 | 98 |
|  | 99 | 74 | 113 | 104 |
|  | 94 | 101 | 89 | - |
| Mean + SEM | **99 + 3** | **94 + 12** | **102 + 4** | **92 + 4** |

**Table 2. The CYP3A activity in control and DSP-4 treated female and male mice.**

| **CYP3A activity (pmol/mg protein/min)** | | | |
| --- | --- | --- | --- |
| **females** | | **males** | |
| control | DSP-4 | control | DSP-4 |
| 447 | 485 | 1069 | 462 |
| 699 | 452 | 754 | 699 |
| 666 | 505 | 837 | - |
| 670 | 647 | 746 | 545 |
| 936 | 627 | 509 | 496 |
| - | 696 | 595 | 284 |
| 566 | 614 | 626 | 218 |
| 539 | 629 | 399 | 267 |
| **646 + 59** | **582 + 31** | **692 + 74** | **424 + 66** |

**Table 3. The pituitary level of GHRH in control and DSP-4 treated female and male mice.**

| **GHRH (pg/mg tissue)** | | | |
| --- | --- | --- | --- |
| **females** | | **males** | |
| control | DSP-4 | control |  |
| 287 | 373 | 201 | 310 |
| 388 | 329 | 215 | 321 |
| 239 | 274 | 234 | 308 |
| 314 | 335 | 279 | 242 |
| 483 | 389 | 285 | 113 |
| 337 | 348 | 366 | 242 |
| 336 | 301 | 403 | 254 |
| 303 | 322 | 337 | 241 |
| **336 + 26** | **334 + 13** | **290 + 26** | **254 + 23** |

**Table 4. The CYP3A activity in control (NET^+/+^) and NET knockout female and male mice.**

| **CYP3A activity (pmol/mg protein/min)** | | | |
| --- | --- | --- | --- |
| **females** | | **males** | |
| control | Knock-out | control | Knock-out |
| 1129 | 598 | 830 | 995 |
| 919 | 926 | 1087 | 950 |
| 788 | 629 | 1156 | 1102 |
| 851 | 848 | 946 | 1081 |
| 759 | 612 | 910 | 879 |
| 958 | 823 | 966 | 779 |
| 1005 | 1233 | 842 | 759 |
| 1078 | 1344 | 972 | 671 |
| 1166 | 945 | 942 | 665 |
| 939 | 971 | - | 872 |
| 1061 | 987 | - | 720 |
| 965 | 1004 | - | 699 |
| **968 + 37** | **910 + 67** | **961 + 35** | **848 + 45** |

**−** not assessed
