# Supplementary figures and images for: The impact of noradrenergic neurotoxin DSP-4 and noradrenaline transporter knockout (NET-KO) on the activity of liver cytochrome P450 3A (CYP3A) in male and female mice
Source: Pharmacol Rep. 2022 Aug 26;74(5):1107–14. doi: 10.1007/s43440-022-00406-8 (PMC9584982; doi:10.1007/s43440-022-00406-8)

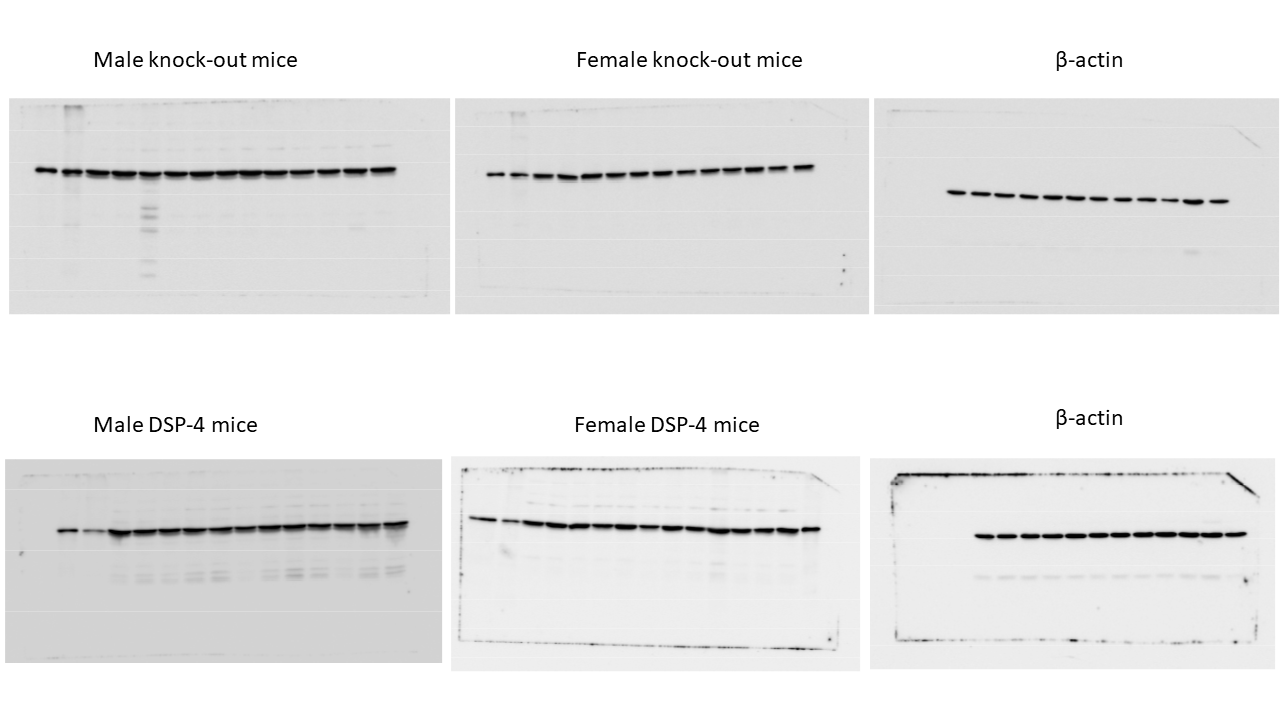

Supplement: Supplementary file 2 — Supplementary file2 (TIF 351 KB) [file 43440_2022_406_MOESM2_ESM.tif]
